# Older Adults’ Conduct of Everyday Life After Bereavement by Suicide: A Qualitative Study

**DOI:** 10.3389/fpsyg.2020.01131

**Published:** 2020-06-19

**Authors:** Lisbeth Hybholt, Lene Lauge Berring, Annette Erlangsen, Elene Fleischer, Jørn Toftegaard, Elin Kristensen, Vibeke Toftegaard, Jenny Havn, Niels Buus

**Affiliations:** ^1^Center for Relationships and De-escalation, Psychiatry Region Zealand, Slagelse, Denmark; ^2^Department of Regional Health Research, University of Southern Denmark, Odense, Denmark; ^3^Danish Research Institute for Suicide Prevention, Mental Health Centre Copenhagen, Capital Region of Denmark, Copenhagen, Denmark; ^4^Department of Mental Health, Johns Hopkins Bloomberg School of Public Health, Baltimore, MD, United States; ^5^Centre for Mental Health Research, Australian National University, Canberra, ACT, Australia; ^6^NEFOS, Network for People Affected by Suicidal Behaviour, Odense, Denmark; ^7^Sydney Nursing School, Faculty of Medicine and Health, University of Sydney, Sydney, NSW, Australia; ^8^St. Vincent’s Hospital, Sydney, NSW, Australia; ^9^St. Vincent’s Private Hospital, Sydney, NSW, Australia

**Keywords:** bereavement, everyday life, older adults, suicide, qualitative research

## Abstract

**Background:**

The loss of a loved one to suicide can be a devastating experience that can have negative long-term effects on the social life and physical and mental health of the bereaved person. Worldwide, an estimated 237 million older adults have experienced suicide bereavement. As assumed in critical psychology, “the conduct of everyday life” reflects the social self-understanding by which people actively organize their lives based on their personal concerns, negotiation with co-participants in various action contexts, and their life interests. Bereaved people may change their social self-understanding as they adjust to their new roles and relationships in everyday life. The aim of this study was to investigate how older adults bereaved by suicide conducted their everyday life during the first 5 years after the loss of a loved one.

**Methods:**

This was a semi-structured qualitative interview study carried out by a research team consisting of co-researchers (older adults aged ≥60 years and bereaved by suicide), professionals, and researchers. The team conducted 15 semi-structured interviews with 20 older adults bereaved by suicide. The interviews were audio-recorded and verbatim transcribed. The participants’ mean age was 67.6 (range 61–79) years at the time of the loss. Data were thematically analyzed through a “conduct of everyday life” theoretical perspective.

**Results:**

We constructed a central theme, “the broken notion of late-life living” in that late-life would no longer be as the participants had imagined. They struggled with their understanding of themselves and other people in social communities when they pursued their concerns adjusting to their broken notions of late-life living. We construed three primary concerns: (1) seeking meaning in the suicide, (2) keeping the memory of the deceased alive, and (3) regaining life despite the loss.

**Conclusion:**

The participants’ bereavement process was influenced by their stage in life. They perceived themselves as having reduced possibilities to restore their life project and limited time to re-orient their life. Age-related factors influenced their possibilities to pursue their concerns in order to adjust to their new life conditions.

## Introduction

It can be a devastating and life-disrupting experience to lose a loved one to suicide, and this may even have negative long-term influences on the bereaved person’s social life and physical and mental health (Pitman et al., 2014; Maple et al., 2017). The past year and lifetime prevalence rates of adults’ exposure to suicide among family, friends, or someone known personally have been estimated at 3.8 and 24.7%, respectively (Andriessen et al., 2017b). In 2017, there were 962 million adults ≥60 years in the world (United Nations, 2017). Assuming that exposure to suicide is similar for older adults, some 37 million older adults may have experienced a suicide in the past year and 237 million during their lifetime. Despite these numbers, older adults bereaved by suicide is an understudied area (Andriessen et al., 2017a), and a systematic search revealed a lack of research about this large group (Hybholt et al., 2018).

Suicide bereavement is associated with an increased risk of prolonged grief, as well as elevated risks of depression, anxiety disorders, psychiatric hospitalization, and death by suicide (Young et al., 2012; Pitman et al., 2014). Although people bereaved by sudden death and suicide experience social awkwardness and attitudes in their social environment, those bereaved by suicide experience greater stigma (Pitman et al., 2018). Stigma is associated with psychosocial distress and a higher risk of suicidal ideation and behavior (Pitman A. L. et al., 2017; Scocco et al., 2017). People bereaved by suicide also have a higher rate of sick leave and disability pension use (Erlangsen et al., 2017). They may also be affected in terms of social functioning, for instance, withdrawing from social interactions and feeling shame, embarrassment, guilt, and the inclination to conceal the death and suppress their emotions and grief (Chapple et al., 2015; Hanschmidt et al., 2016; Peters et al., 2016; Pitman et al., 2016). Uncomfortable feelings such as blame, guilt, and emptiness are enhanced by the inability to find meaning in a death by suicide (Shields et al., 2017). Additionally, young adults bereaved by suicide report a lack of informal support or delays in receiving support compared to those bereaved by sudden death (Pitman A. et al., 2017).

According to the dual-process model of coping with bereavement, bereavement is both an inner psychological condition and a social and cultural event. Bereavement may change a person’s identity and self-understanding as they work on their new roles and relationships in everyday life (Stroebe and Schut, 1999). Among older adults, grief may be affected by age-related difficulties in everyday life, such as reduced/limited social interaction or challenges in restoration-oriented tasks (Hansson and Stroebe, 2007). Bereaved older adults experience higher levels of emotional and social loneliness and are at increased risk of suicide (Shah and Meeks, 2012). However, older adults also seem better prepared to cope with bereavement compared to younger age groups because they are more experienced with death and have higher emotional control (Hansson and Stroebe, 2007). People bereaved by suicide might handle the situation differently depending on their age and prior experiences with death; mature individuals who have experienced several life events receive comfort through open dialogue, while younger individuals handle difficult situations by concealing their emotions and suffering (Kasahara-Kiritani et al., 2017).

### Theoretical Perspective: Conduct of Everyday Life

Losing a significant other to suicide is likely to affect everyday life on a long-term basis. In critical psychology, the notion of “the conduct of everyday life” encapsulates the actions and social self-understanding by which individuals actively organize their daily life. This is based on their personal concerns, negotiation with co-participants in various action contexts, and their life interests. The conduct of everyday life must be understood from the individual’s first-person perspective (Dreier, 2008, 2011; Holzkamp, 2013, 2016; Scraube and Højholt, 2016). Everyday life is conducted with meaningful regular activities that re-occur in a seemingly repetitive manner but without actually being repetitions since the circumstances change. Daily routines such as eating breakfast are taken for granted until they are disrupted, for example, by an illness or the loss of a loved one. A person’s everyday life changes many times over a lifetime with events like retirement or becoming a grandparent. Changes can be more or less profound, but when life conditions change, it may initiate substantial identity work in coming to understand oneself, other people, and collective subjectivity in the light of the new life conditions (e.g., bereaved by suicide). This subsequently influences the person’s conduct of everyday life. Personal concerns refer to what really matters for an individual. An individual may pursue personal concerns across the various action contexts in their entire social practice. Action contexts are anchored in time, space, and contexts in everyday life where participants come together (e.g., family members mourning at a ritual). Action contexts are characterized by the confluence of the participants’ common, differing, and conflicting concerns, and it can be difficult to find ways to pursue one’s personal concerns (jointly, allied with, and in opposition to others). Thus, a person’s scope of action possibilities is not solely up to the individual; it also depends on other people’s activities and actions. People sometimes give up pursuing a concern and comply with circumstances if it is in the person’s life interest. Life interests are the basic human interest to sustain and expand the influence on one’s life conditions and life quality.

The aim of this study was to investigate how people bereaved by suicide at age ≥60 conducted their everyday lives in the first 5 years following the loss of a loved one.

## Materials and Methods

A group consisting of professionals, researchers, and older adults bereaved by suicide collaborated to explore everyday life experiences after a loss. The group co-created a qualitative semi-structured guide to interview older adults bereaved by suicide.

### Study Setting

The interviews were carried out in Denmark from September 2017 to July 2018. There is a free-of-charge public health care system for all citizens in Denmark, including free access to general practitioners (GPs). A specific care plan for people bereaved by suicide is not available, but GPs can prescribe subsidized psychotherapy, thus, reducing therapist fees by 40%. Older adults bereaved by suicide might not contact their GP in relation to bereavement. In Denmark, there are two, not-for-profit non-governmental organizations (NGOs) offering support to people bereaved by suicide. The Network for the Affected by Suicidal Behaviour (NEFOS) offers home-based support to older adults bereaved by suicide based on the clinical assumption that this group is less inclined to seek help and therefore may face obstacles attending appointments outside the home (Erlangsen and Fleischer, 2017). The other NGO is the National Association for the Bereaved by Suicide, which offers peer support meetings as well as “walk and talk” events.

The interviews were part of a Co-operative Inquiry into the need for psychosocial support among the elderly bereaved by suicide with the overall aim to develop and test new psychosocial interventions (Hybholt et al., 2019). Co-operative Inquiry research is “with” people rather than “on” people, so the production of knowledge is a joint venture among an array of stakeholders who work together to create relevant and practice-oriented knowledge. This is achieved in four research steps: preparation, orientation, intervention, and evaluation (Heron, 1996; Reason and Bradbury, 2008; Berring et al., 2016). The interview study was a part of the orientation step, where the goal is to gain a deeper understanding of the subject under research. We interviewed older adults about their everyday lives and experiences with the purpose of identifying needs for psychosocial support among older adults who experience suicide bereavement. In this paper, we focused on the research question: “How do elderly persons bereaved by suicide experience their everyday life after the death of a loved one?”

### Participants and Recruitment

Recruitment was based on a purposive sampling designed to create a maximum variety of habitat, gender, kinship, and civil status (Patton, 2015). We exclusively included participants who had lost to suicide within the previous 5 years. This decision was made to ensure that we learned about the participants’ interactions with present day health care and social care services. Participants were recruited through the two NGOs; the mental health services in two regional health care providers in Denmark; and notices about the project at relevant meetings, websites, conferences, and on social media. None of the people who contacted the principal investigator for further information about the study refused to participate.

A total of 15 semi-structured single interviews with 20 participants were conducted across Denmark. We conducted five of the interviews with married couples. For some of the participants (*n* = 7), it was an unexpected death. Others (*n* = 4) became aware of the risk of suicide weeks or months before it happened, and some (*n* = 9) had lived with worries about suicide for years or decades due to the deceased’s previous suicide attempts, severe mental illness, or other problems. The participants were 7 men and 13 women aged between 61 and 79 years at the time of the loss (mean: 67.6 years). The time between the death by suicide and the interview ranged from 7 to 66 months (mean: 25.2 months). The kinship to the deceased was parent (*n* = 9), stepparent (*n* = 1), child (*n* = 1), child-in-law (*n* = 1), spouse (*n* = 4), parent-in-law (*n* = 1), and grandparent (*n* = 3). Seventeen of the participants had retired from work, and seven lived alone (see Table 1).

**TABLE 1:** Participants.

Participant number	Age at time of loss in age intervals	Gender	Living with a partner	Kinship to deceased	The time between the death and the interview in month	Contact to an not-for-profit non-governmental organization (NGO) in relation to the loss	Retired from work at time of the interview	Interview with spouse
1	60–64	Male	Yes	Father	14	Yes	Yes	Yes
2	60–64	Female	yes	Mother	14	Yes	Yes	Yes
3	60–64	Female	No	Mother	23	Yes	Yes	No
4	65–69	Female	No	Spouse	66	Yes	Yes	No
5	65–69	Female	No	Spouse	44	Yes	Yes	No
6	70–79	Male	Yes	Father-in-law	23	No	Yes	No
7	70–79	Female	No	Grandmother	12	No	Yes	No
8	65–69	Male	Yes	Son	11	No	Yes	Yes
9	65–69	Female	Yes	Daughter-in-law	11	No	Yes	Yes
10	65–69	Female	Yes	Mother	7	Yes	Yes	Yes
11	65–69	Male	Yes	Stepfather	7	No	No	Yes
12	65–69	Female	Yes	Mother	40	Yes	No	Yes
13	60–64	Male	Yes	Father	40	Yes	No	Yes
14	65–69	Female	No	Spouse	11	No	Yes	No
15	60–64	Female	Yes	Mother	39	No	Yes	No
16	60–64	Male	No	Spouse	24	Yes	Yes	No
17	70–79	Female	Yes	Grandmother	23	No	Yes	Yes
18	70–79	Male	Yes	Grandfather	23	No	Yes	Yes
19	65–69	Female	Yes	Mother	27	Yes	Yes	No
20	70–79	Female	No	Mother	45	Yes	Yes	No

Eleven participants had previously contacted the mentioned NGOs and had received support from them prior to the interview. The participants had had varying degrees of contact with professionals including police officers, general practitioners, psychologists, psychiatrists, and nurses in relation to the suicide death.

### Interviews

A semi-structured interview guide was developed (Kvale and Brinkmann, 2014) in a collaborative working group in which the members had different areas of expertise. Older adults bereaved by suicide and professionals were experts based on their lived experience, while the researchers were academic experts in developing a qualitative interview guide with open questions. The interview guide consisted of four themes exploring: (1) what had happened in the participant’s life since the loss (questions about their own thoughts and actions and their experience of other people’s reactions including professionals, friends, acquaintances, and family members), (2) how they lived their everyday life, (3) their reflections on how their age might have influenced their everyday life, and 4) what kind of support they felt would have helped/could help them. All interviewers were very familiar with the interview guide. The project investigator had the task to provide the formal information, and the co-researcher would ask the first opening question in theme one. Thereafter, we emphasized to have a dialogue guided by the themes in the interview guide with no prior agreement about who asked the specific questions (see Supplementary Material).

Each interview was conducted by two interviewers: a Ph.D. educated female researcher (principal investigator LH) and one co-researcher. The project investigator had expertise with conducting qualitative interview about sensitive personal issues and is an expert within everyday life and learning processes. The co-researchers were retired older adults with lived experience as bereaved by suicide [JT (male), VT (female), and EK (female)]. The interviews were conducted with the sole presence of the participants and the two researchers. As they were not familiar with each other, the interview situation was initiated by a presentation of the researcher and the co-researcher and their academic/personal background. The interviews were carried out in the participants’ homes, except for one person who preferred meeting in an office provided by the research team. The interviews were audio-recorded and verbatim transcribed. The mean length of the interviews was 109.5 (range: 81–134) min. After each interview, the principal investigator made field notes with the impression of the interview and setting. The participants were offered to read the transcript. Two participants accepted the offer and had a few elaborations of the content. When we were unable to identify new issues during several interviews, we decided to end the data generation.

### Analysis Strategy

All interviews were analyzed thematically (Coffey and Atkinson, 1996) with the conduct of everyday life as the theoretical perspective (Dreier, 2008, 2011; Holzkamp, 2013, 2016; Hybholt, 2015; Scraube and Højholt, 2016). The analysis was divided into seven steps. Four research teams consisting of a researcher and co-researcher carried out the first three steps of the analysis. The principal investigator, LH, conducted the last four steps in collaboration with LLB and NB, which was supported and challenged by continual dialogue with the entire research group. The seven steps were:

(1) Becoming familiar with the data: each research team read and re-read a designated portion of the interview transcripts including a short description of the interview/setting based on the field notes while noting their first impressions.
(2) Generating initial codes: each research team separately created open coding of the interviews.
(3) Describing categories in each interview: each research team explored their different views on what was at stake in the participants’ everyday life. They described their perspectives on the interview in categories including codes and selected citations from the interview to consolidate the category.
(4) Theorizing everyday life as bereaved by suicide: scrutinizing the conduct of everyday life in interview transcripts, notes, and categories for each participant asking central theoretical questions such as: How did the loss disrupt the conduct of everyday life? What were their primary concerns after the bereavement? How did they pursue their concerns in various social interactions? How did social interactions influence their pursuit of concerns? What were their reasons to act as they did in relation to their concerns? How did they come to understand themselves, other people, and collective subjectivity in various action contexts?
(5) Patterns and concerns across all data material: the principal investigator wrote memos as part of an iterative analytical process between the theoretical analysis and the issue under research (everyday life among older adults bereaved by suicide). This analytical step was designed to explore how old age influenced the conduct of everyday life when bereaved by suicide. Through theoretical interpretation of patterns in the interviews, we developed an overarching central theme that provided an abstract understanding of the new life condition as an older adult bereaved by suicide. The central theme was the standpoint, which reasoned the conduct of everyday life seen from the older adults’ perspective. We identified the most important concerns pursued in their everyday life while adjusting to late-life living in the context of a loss to suicide. There were three primary concerns that older adults bereaved by suicide simultaneously pursued in different ways in their daily struggle of adjusting to understand themselves and others in the social context of their new life condition.
(6) Challenging the emerging interpretation: the authors (LH, NB, LLB) read, re-read, and discussed the emerging interpretations and reviewed the data to examine outliers and develop thematic patterns. All authors of the paper were engaged in an ongoing written and face-to-face dialogue to discuss the preliminary findings.
(7) Writing up the findings by condensing the main issues in the central theme and how they govern the conduct of everyday life and the pursuit of concerns for older adults bereaved by suicide.

### Ethics

In accordance with Danish legislation, the regional research ethics committee and the Danish Data Protection Agency (REG-082-2017) were notified about the interview study; neither institution had any reservations about the protocol. All participants gave written informed consent in accordance with the Declaration of Helsinki II. Interview responses were kept fully confidential, and all details that could potentially be used to identify individual participants were altered in the presented excerpts. Data were kept in a secured database. A responsive safety plan for the participants and interviewers ensured regular group supervision of the interviewers and professional counseling for the participants if needed.

## Findings

Based on the analyses of the interviews, we developed a central theme of “the broken notion of late-life living.” The central theme was an abstract interpretation of the interviews as a whole, therefore, we decided to exemplify the theme in depth by including lengthy quotes from a single case to contextualize the theme. We also noted that older adults strived to adjust to their broken notion of late life in their conduct of everyday life, and three primary concerns emerged: (1) seeking meaning in the suicide, (2) keeping the memory of the deceased alive, and (3) regaining life despite the loss.

### The Broken Notion of Late-Life Living

Death by suicide signified not only the loss of a loved one but also the loss of the older adults’ notion of late-life living, as life would no longer be as they had imagined. The broken notion of late-life living could be losing the possibility of being grandparents if the deceased was their only offspring and childless or if they were cut off from contact with grandchildren. It could also imply the loss of an imagined life as surplus parents/grandparents because the bereavement had taken their resources or they had to take responsibility for other family members (either taking care of their condition or compensating for the deceased person). It could also be the loss of living a planned, pleasurable late life as a retired couple, either because the spouse died by suicide or because a spouse was strongly affected by grief.

One example of how the notion of broken late-life living influenced the conduct of everyday life was provided by a married couple (aged 66 and 69) who had lost their son 40 months earlier. Since their son was a child, they had worried about his mental wellbeing and had substantially supported him until his death by suicide when in his 30s. They were aware that their son was suicidal as he had made suicide attempts previous and had expressed suicidal ideations. The mother discussed the loss of what was supposed to have been a part of her and her husband’s late-life living:

*I often think of the fact that we will have no more grandchildren. We will have no more grandchildren, and our friends will continue to have grandchildren, and you feel a stab in your heart when hearing that someone has a new grandson, Otto, another Arthur, and whatever else they name them these days [*…*] It is positive that we already have two grandchildren, but we would have liked a little [name of deceased* son]… […] And there will be no new pictures of him, because of the situation – which is fixed – that is very strange. I think about it, and I *know you do too (addresses husband) [*…*].* (Participant 12)

The conduct of everyday life reflects the actions and social self-understanding by which individuals actively organize their everyday life based on personal concerns. The parents had a shared concern for their son’s wellbeing and had organized their lives to support him. They wanted to see him thrive and eventually settle down with a family of his own. However, his death had terminated their son’s path through life and painfully accentuated their loss of a lifelong concern. As a result, the notion of the imagined late life with their son was broken, and this was particularly profound because the probability of substituting or rebuilding their life project was limited. They would never have a grandchild from their deceased son.

It also left them in a situation where the mother had to struggle to support both her own and her husband’s continued existence. The husband had lost meaningful direction in life, as he could no longer pursue the concern as a supportive father for his son. In the following quote, the husband talks about memorable situations with their son, which reminds the wife of her loss of a content husband:

*Father: When looking at the memorial we have on Facebook, you see [name of deceased son], and you can see those football pictures from the matches which is EXACTLY when he was happy. It was so him – it was [name of deceased son]. I like seeing the pictures where he*… *him in the football stadium (tearful voice).*

*Mother: Yes, where he was happy, and you were happy.* (Participant 12, Participant 13)

From the mother’s perspective, she had not only lost her son and his participation in her imagined later life, she had also lost her well-functioning, happy husband. As the conduct of everyday life is negotiated in interaction with co-participants, her husband’s condition significantly impacted her daily life. Their son’s suicide had replaced their shared concern for his wellbeing with a daily struggle for their own existence. It was in the wife’s interest to be concerned about them both and their adjustment to their broken notion of late-life living. She revealed that she would not be able to bear to lose him as well. Furthermore, she also wanted to resume the ability to fulfill her role as a useful mother and grandmother, as illustrated in the following quote:

*I have become fairly sensitive and sometimes I need to take in those antennas [brings her hands to her head] and think, and then sometimes I also sort of need to say to [name of deceased], “You need to leave now,” because there needs to be room for the other children and grandchildren as well. There is no doubt that I am not the same person; I am certain of that.* (Participant 12)

Social self-understanding is the process through which people perceive themselves and others perceive them in different action contexts. The death by suicide changed the mother’s social self-understanding; she no longer experienced herself as the same strong person. Her decreased health affected her possibilities to act as wanted toward her remaining descendants. Thus, her notion of late-life living was broken, and her altered state meant that her personal concern of motherhood was no longer natural. Indeed, it had become a struggle in her conduct of everyday life with her other children and grandchildren. Furthermore, the couple experienced that other people did not understand how important their deceased son was for them in their conduct of everyday life. As an example, a friend directly told her to move on and stop talking about her dead son. Both felt that other people perceived them as obsessed with their late son. This made the broken notion of late-life living even more profound, as their participation in everyday life while adjusting to their situation distanced them from some of the people they used to view as close relatives or friends, which shrunk their social network.

### Concerns in Everyday Life

To adjust to the broken notion of late-life living, the participants pursued three concurrent concerns: (1) seeking meaning in the suicide, (2) keeping the memory of the deceased alive, and (3) regaining life despite the loss. The following sections elaborate how the participants pursued the concerns in their conduct of everyday life.

#### Seeking Meaning in the Suicide

Seeking meaning in the suicide involved the bereaved person trying to understand why their loved ones wanted to die. From the older adults’ first-person perspective, it was crucial for their adjustment to their broken notion of late-life living to comprehend the suicide of their loved one. They pursued this concern by scrutinizing problematic events in their everyday life with the deceased, such as divorce in the family and lack of parental involvement during childhood. They reviewed their own and others’ roles in the suicide death and dissected the deceased’s condition, actions, and statements in the months and years before the suicide. They ruminated over signs that seemed obvious in hindsight. They searched for information about the life of the deceased through dialogue with people in close contact with them. This retrospective inquiry was often combined with seeking general knowledge about suicidal behavior or mental disorders through professionals or literature. Pursuing the meaning-seeking concern completely consumed some participants’ mental activity. Still they resumed daily activities such as housekeeping, leisure activities, and volunteer work, which could lead to other people praising them for coping well. However, from their perspective, nothing was the same, regardless of seemingly returning to regular activities.

Some participants comprehended the deceased’s difficulties and pain if the deceased had suffered from severe mental illness. The participants expressed this in statements such as: “He got peace and, actually, I also got peace,” *(Participant 7), “*Her fear of life was greater than her fear of death,” *(Participant 15)* or *“*Now he is a star in the sky. It is as it is meant to be.” *(Participant 12).* Still they ruminated and grieved. Others kept seeking meaning to attempt to adjust to their broken notion of late-life living. For example, a woman, aged 72, whose husband unexpectedly took his life 5 years earlier:

*I’m so sorry on behalf of my granddaughter because she*… *none of us are able to forgive him, maybe one day*… *You will feel better yourself if you are able to forgive*… *but we think he should have come to us*… *if we just had noticed that there was something wrong. But we had no problems, so I didn’t think*… *we had a house and a summer house and*… *we had no problems. We were very happy together. we did not have any financial problems or anything.*… *I have visited a clairvoyant twice*… *What I wish to know is why he did it, but he doesn’t really want to talk about it, the clairvoyant says. so I will go back to ask again and see if I might learn more.* (Participant 4)

This bereaved spouse was convinced it would be in her own and her granddaughter’s best interest to be able to forgive the deceased. It had not been possible because they were unable to understand why he died by suicide. She pursued this concern by planning a third visit to a clairvoyant, even though her late husband had refused to answer the “Why?” question at earlier afterlife meetings. Still, she wanted him to answer, convinced that it could provide some kind of meaning that would enable her and her granddaughter to forgive, let go, and move on.

Striving to find meaning was part of coming to understand themselves as a person bereaved by suicide, as well as their own and other people’s roles in the suicide, and the deceased’s choice to die. Finding meaning could lead to gradually comprehending or accepting the meaninglessness of death by suicide, which could extend the older adult’s scope of possibilities for repairing and settling with their broken notion of late-life living.

#### Keeping the Memory of the Deceased Alive

Concern about keeping the memory of the deceased alive required integrating the deceased into the participant’s conduct of everyday life. It was crucial that the deceased was not taken out of history as if they had never existed, but instead continued to impact their own life and those of significant others. The participants kept their memory alive by incorporating regular memorial activities into daily living at home, such as talking to a picture of the deceased or lighting a candle. They also pursued the concern outside the home at various formal occasions; for example, by mentioning the deceased in speeches, ritual visits at the graveyard, or celebrating the deceased’s birthday with relatives and friends. Others shared stories and knowledge about the deceased, ensuring that their history propagated. Doing so revealed their loss and huge sorrow, and participants often felt that others did not share or understand their concern. Instead, they felt that most people avoided talking about the deceased, told them to stop grieving, or said that the deceased had acted cowardly or indecently. Some of the participants found it unbearable to exclude their late loved one from their conduct of everyday life. Avoidance by others could make it an individual pursuit, which made it difficult to address.

One mother, aged 64, had lost her only child 23 months earlier. Her daughter had been mentally ill for years, and she was aware the daughter was suicidal. In her apartment, she had many pictures and a large painting of the daughter as daily reminders. She did not only lose her daughter; her daughter was married to a man with children from an earlier relationship, and he now denied her to have contact with them, thus she lost them as well. Except for a close friend of her daughter, she was the only one pursuing the concern of keeping the memory of her daughter alive. In the following quote, she told us about a family gathering:

*I still really miss*… *The fact that we do not talk about her*… *To me, that is the worst part. We just had a cousin’s reunion. At the reunion no one brought up her name or*… *no. Not at all*… *and that made me so sad.* (Participant 3)

As the conduct of everyday life is an ongoing negotiation with co-participants, it influences older adults’ options for keeping the memory of the deceased alive when other people avoid mentioning or talking about the deceased. Most participants remained silent to avoid bothering other people with their concern. More than half opted to participate in activities provided by the two non-profit organizations who offered professional counseling and peer support to people bereaved by suicide. At those sessions, they could experience mutual understanding and support for their concerns among peers and professionals. As a man who lost his wife suddenly said: “When I’m with the non-bereaved I feel awkward taking about my wife, but among others bereaved by suicide, I’m like a fish in the water.” *(Participant number 16)*. Seen from the participants’ first-person perspective, it created a “them and us” that made the broken notion of late-life living even more profound. However, it was more fulfilling to share the concern with people who personally knew the deceased and thus could contribute personal knowledge and shared sorrow and memories.

Keeping the memory of the deceased alive was a way for the participants to adjust to their broken notion of late-life living; they transformed their connection to the deceased into activities dedicated to keep their loved one alive in everyday life. It was a struggle for participants to establish new memorial activities because it exacerbated their sorrow and reminded them of lost late-life possibilities. In addition, other people negatively affected their ability to keep the memory of the deceased alive, as they often were not supportive.

#### Regaining Life Despite the Loss

The concern of regaining life despite loss was characterized by organizing everyday life by balancing mourning and taking part in daily living (i.e., not mourning). An example of taking time to mourn was talking with people who had the ability to be present with someone who was grieving. Some lacked confidants due to illness, death, or age restrictions, which reduced possibilities to mourn. Others participated in grief groups or voluntary organizations for those bereaved by suicide. Some participants had to prioritize caretaking activities for family members who needed their attention. They felt that fulfilling their obligation for a loved one’s wellbeing helped them briefly set aside their own grief. Some had relatives who strongly encouraged them to participate in familiar activities and recognized that this was helpful. Other participants organized breaks from grieving by filling their calendar. Although the activities had lost some meaning due to the participants’ overshadowing grief, they also helped normalize life despite the loss. One mother who had unexpectedly lost her only son 7 months earlier threw herself into housework, even though she had severe hip pain and was waiting for surgery:

*I have really cleaned! ALL bookcases. Every corner, the bedroom closet, the dressers. I have practically taken apart the kitchen and washed it down. I set myself the goal to stay busy, in particular when my husband was out. I said to myself: “You do this now,” and then I began*…. *I do not think it is wise to sit down in a chair and think, “Poor me.” You need to try to pull yourself up by the bootstraps and begin doing something [*…*]. It also helps you feel better; I believe, to kick yourself into gear and steer your thoughts to*… *something else. It has helped me. I enjoy cleaning and tidying up – but this time it was just on a larger scale. Because simply sitting and doing nothing does not help you. It really does not.* (Participant 10)

This mother attempted to regain her life despite the loss, using exaggerated housekeeping as a way to survive the intense grief and reasoned that it was a way to achieve good late-life living. Heavy cleaning had been effective during an earlier life crisis, and she overcame the struggle based on her conviction that it would help her regain life as a joyful wife, resourceful mother-in-law, and dedicated grandmother. She wanted to be useful to her loved ones. She also conducted her everyday life with breaks to mourn. Some participants revealed that it could be more troublesome to create breaks from grief if they had physical challenges; e.g., preventing them from doing household chores or participating in activities outside the home.

The participants also pursued the concern of regaining life by considering other people’s expectations of them to finish grieving and move on. They continued life in a seemingly usual way, but from their perspective, they were in a grieving darkness and dealing with a new life condition that changed everything. They concealed their grief as they wanted to fit social conventions, did not want to bother other people, and felt it was not appropriate to reveal their sorrow.

It could be difficult to create breaks from grief because they lacked obligations such as returning to work or family members depending on them. Furthermore, it could be hard to implement previously successful strategies like vigorous cleaning due to age-related factors like reduced physical strength, lack of endurance, or immobilization. This concern was part of adjusting to late-life living that would never be as they had imagined, but it still seemed possible to live a substantial life despite the loss.

## Discussion

The main theme: “*the broken notion of late-life living*” provided the insight that the participants considered their chances of substituting or rebuilding their life goals as limited. For instance, it was regarded as too late in life to rebuild the family with a new child or to find a partner functioning as a life witness. The Socioemotional Selectivity Theory suggests that time left to live influences our perspective in life. Older people describe their future as limited and are aware that they do not have much time left to pursue their goals (Carstensen et al., 1999; Charles and Carstensen, 2010). This was supported by a study exploring older adults’ (age 60–83 years) experiences of their aging in relation to concerns about everyday life. The older adults contemplated their limited time left and their imminent death, which created an experience of time pressure to archive personal hopes and desires (e.g., being alive to see offspring grow up). The awareness of restricted time left was experienced as limiting the potential fulfillment of future possibilities and made them concerned about the futures of loved ones after their death (Russo-Netzer and Littman-Ovadia, 2019). The main theme in the current study indicated that suicide death profoundly shapes the remaining future for older adults bereaved by suicide. Their original expectations and wishes for late-life were broken. They did not simply experience time pressure to achieve personal hopes and desires. In addition, they struggled to pursue new concerns in their conduct of everyday life to be able to adjust to their new life condition. Furthermore, some participants took responsibility for other family members either by taking care of them or compensating for the deceased person. Their position changed with new responsibility, as others now depended on them. The awareness of a limited future can be considered a natural part of getting older, but forced re-orientation in life and increased liability may intensify time pressure for older adults bereaved by suicide.

The changes in the bereaved older adults’ late-life living can be understood as a major “biographical disruption.” This concept was originally developed in relation to chronic illness, as a critical situation that disrupts individuals’ structures of everyday life, taken-for-granted assumptions, and expectations and plans for the future. As a result, individuals require a biographical repair to make sense of their remaining life, restore normality and control, and find new meaning and identity (Bury, 1982; Locock and Ziébland, 2015). Owens et al. (2008) used the concept to understand the experiences of parents bereaved by suicide. They found that participants’ life work as parents was profoundly disrupted by the suicide. They attempted to repair their own and their children’s biographical disruption, and defend everyone’s moral reputation. According to Bury (1982), the attempt to normalize in the face of disruption is a part of the repair work. This hypothesis is supported by a study of older adults (age 73–91 years) with multiple chronic conditions that limited their daily living. They learned to live with their disabling condition by accepting it as an unavoidable and normal aspect of aging (Clarke and Bennett, 2013). The participants in the present study struggled to find meaning with the suicide deaths, and they often experienced their situation as strange when in social contact with others. The importance of normalization as a part of the repair work might explain why many subjects felt the need to participate in communities with peers. Russo-Netzer and Littman-Ovadia (2019) stated that connections and the feeling of belonging to a group were secure anchors for older adults that were important for coping with late-life challenges. Bereavement by suicide seems to jeopardize older adults’ social connections and feelings of belonging. It may amplify their struggle to adjust to new life conditions, making them feel that the possibilities for normalization in everyday life are limited.

### Strengths and Limitations

A key strength of this study was the ability to obtain in-depth understanding of the experiences, needs, and everyday life of older adults bereaved by suicide, which is an understudied area of research. Furthermore, we succeeded in recruiting nine hard-to-reach research participants who had not attended any support groups or other NGO supports related to suicide bereavement. Moreover, it is important that all authors contributed with different expertise and perspectives, which helped validate the findings.

The peer interviewers experienced a special connection with the participants, as they related to each other based on their shared experiences. The peer interviewers believed that their presence made it easier for the participants to express themselves. However, this belief is based on the perspective of the peer interviewers and needs further research to be validated, such as linguistic analysis of the actual verbal interactions in the interviews. As in a study (Forbat and Hubbard, 2016) where previous careers of people receiving palliative services interviewed current careers, it is possible that the interviewees would change the conversation topic when the peer interviewer shared their experiences, thus decreasing the opportunities for elaboration and disclosure of the investigated topic. We prompted the peer interviewers to focus on the participants’ experiences. We agreed that the project investigator, with her outsider position, had the primary responsibility for covering the themes in the interview guide. Still, the peer interviewers might have influenced the interviewees in a direction aligned with their own key issues.

The interview with participant eight and participant nine, who had lost their mother/mother in law, were outliers from the central theme. They pursued the identified concerns for a short period and then continued their everyday life as before. The suicide of an older family member did not disrupt their notion of late-life living. For the purpose of research categorization, Cerel et al. (2014) suggested a nomenclature that differentiated the bereaved by suicide on a nested continuum, from a larger population “exposed” to suicide to the smallest “suicide-bereaved long-term” group that struggles across a longer period with clinically significant responses to the loss. Thus, it might have been more relevant to differentiate among the types of suicide bereaved and not only age when recruiting participants. Alternative interpretive perspectives could have been included in trying to explain the outliers, such as coping styles, social supports, or attachment styles.

Our goal was a purposive sampling to achieve maximum variety in relation to habitat, gender, kinship, and civil status. It consisted of numerous women and parents, and we did not recruit close friends or siblings. Finally, interpreting the data set through a “conduct of everyday life” perspective allowed particular aspects of the participants’ experiences to stand out, while others moved into the background. Although we believe that this perspective allowed us to study and emphasize important issues in the participants’ lives, an alternative perspective might also have been fruitful in analyzing and reporting findings.

## Conclusion

We have tried to make a contribution to an area with limited research by investigating the conduct of everyday life among bereaved older adults. Participants’ bereavement process was influenced by their stage in life. Their experiences were framed by a broken notion of late-life living, where they perceived themselves as having reduced possibilities to restore their life project and limited time to re-orient their life. Furthermore, we identified age-related factors that influenced the older adults’ possibilities to pursue their concerns in order to adjust to their new life condition, including retirement (reduced social interaction, lack of structure, and obligations), reduced network (e.g., loss of friends to sickness or death), and decreased physical and mental health. These factors may be important to consider when supporting older adults adjusting to their new life following bereavement due to suicide.

## Data Availability Statement

The datasets for this article are not publicly available because of patient confidentiality, participant privacy, and ethical data protection laws. Requests to access the datasets should be directed to LH, Lihy@regionsjaelland.dk.

## Ethics Statement

The project was assessed by the Ethical Committee for Region Zealand in Denmark and is journalized with the journal number: J.nr. 17-000048. Participants gave written informed consent, interview responses were kept fully confidential, and all details that could potentially be used to identify individual participants were altered in the presented excerpts which is in accordance with the Declaration of Helsinki.

## Author Contributions

JT, EK, VT, and LH conducted the interviews. JT, EF, EK, VT, LB, NB, and LH analyzed the interviews. LH drafted the manuscript. AE, LB, and NB revised the draft critically for important intellectual content. All authors contributed to the design of the study and agreed with the final draft.

## Conflict of Interest

The authors declare that the research was conducted in the absence of any commercial or financial relationships that could be construed as a potential conflict of interest.
