# Supplementary material for: Older Adults’ Conduct of Everyday Life After Bereavement by Suicide: A Qualitative Study
Source: Front Psychol. 2020 Jun 19;11:1131. doi: 10.3389/fpsyg.2020.01131 (PMC7316951; doi:10.3389/fpsyg.2020.01131)
Supplement: Supplementary file 1 [file Data_Sheet_1.pdf]

The semi-structured interview guide: **Older Adults' Conduct of Everyday Life after Bereavement by Suicide: A Qualitative Study**

Introduction
[Both interviewers introduce themselves and their roles in the project, the peer-researcher explicitly discloses his/her position.]

PI provides formal information on:

- The objective, design, and purpose of the project
- Benefits and risks of participation
- How we plan to conduct the interview (guide, timeframe, dialogue, breaks)
- Audio recording and anonymity
- The right to withdraw consent.

[The interviewee signs the consent form.]

Check-in question: Is it okay for you to continue the conversation and begin the interview?

Establishing the space for 'intense listening'
[Remember to dwell on what is said by using the person's words, count slowly to five before talking, tolerate silences, so we do not interrupt. Remember to be patient: we have time to listen.
Notice the words the interviewee uses to describe the death and adopt these words in the ongoing interview.]

Theme 1: Exploring the course of events
Introduction: We would like to learn more about your experiences about what happened for you when [name] died, for example, the people you met and the situations you had to deal with.

Question: You said it has been x years/month since [name] died by [words of the interviewee], how are you now?
Question: How did you find out that [name] had died [using the words of the interviewee?]
Question: How did you experience the people you met? What did they say and do? (probe for police officers, undertaker, priest, health care staff, financial advisor, probate court staff).
Question: Which events did you go through? How did you experience (informing others about the death, identification of the deceased, funeral, meeting people afterward).
Question: Who do you usually get support from in difficult situations?
Question: Whom and what did you have around you who could support you? How did you experience their response?

Theme 2: Everyday life as bereaved by suicide
Introduction: We would also like to learn from your experiences of how the loss of [name] influenced your everyday life, tasks, social contacts, roles, expectations, etc.

Question: How do you think the loss of [name] has influenced your everyday life?

Question: How do you think the loss of [name] affected your relationships with other people?
Question: When you think of your family and friends before and after the loss of [name], how do you think your role has changed?
Additional questions: Is there something you do more or less of now? How do you manage your everyday life? Are there things that are difficult to handle, for instance; finances, practical activities, social events, responsibility for others?

Theme 3: Exploring the possible significance of being bereaved at +60
Introduction: In the research group, we have talked about whether age influences the situation when you are bereaved by suicide and we would like to hear your thoughts about this.

Questions: Earlier you mentioned [events, people, everyday life] how do you think your age influenced that [events, people, everyday life]
Question: Some people say that when you are older, your grief and mourning after [words of the interviewee] is taken less seriously, compared to if you were younger. What do you think about that idea?
Question: Some people say that when you are older and experience [words of the interviewee], it may change the perception of your own death and mortality. What do you think about that idea?
Question: We have considered whether it might be of significance if you have experienced loss previously. What is your experience?

Theme 4: Reflections on help and support
Introduction: We would like to hear your thoughts on help and support. This can include things you have experienced as positive or negative. It could also include your suggestions for improvement.

Question: Earlier you mentioned [events, people, everyday life]. How do you think that affected you in terms of support and help, or lack thereof?
Question: If you look back at the time when [name] died, what do you wish had been done to help you?
Question: In your current situation, is there anything you can imagine that would be good for you?

Close [no later than 90 minutes into the conversation]
Introduction: We are about to reach the final part of our conversation. We appreciate your contributions to the study. They will be very helpful in our further work.

Question: Is there anything that you would like to add?
Question: How was it for you to partake in the interview?
Question: Was there anything particularly difficult for you to talk about?
Question: How do you feel now?

[Organise date and time for phone follow-up with PI. Provide contact details for a professional counselor that can be contacted for free if needed. Emphasize that the interviewee can contact the PI if they have any comments or questions regarding the interview or the project.]
